# Investigation of nanotopography on SOCE mediated cell migration via live-cell Imaging on opaque implant surface

**DOI:** 10.1186/s12951-023-02249-8

**Published:** 2023-12-08

**Authors:** Yan Zhang, Kai Li, Guangwen Li, Yazheng Wang, Yide He, Wen Song, Yumei Zhang

**Affiliations:** 1 State Key Laboratory of Oral & Maxillofacial Reconstruction and Regeneration, National Clinical Research Center for Oral Diseases, Shaanxi Key Laboratory of Stomatology, Department of Prosthodontics, School of Stomatology, The Fourth Military Medical University, Xi’an, Shaanxi 710032 China; 2 Department of stomatology, The 986th Air Force Hospital, Xijing Hospital, The Fourth Military Medical University, Xi’an, Shaanxi 710032 China; 3 State Key Laboratory of Oral & Maxillofacial Reconstruction and Regeneration, National Clinical Research Center for Oral Diseases, Shaanxi International Joint Research Center for Oral Diseases, Department of Periodontology, School of Stomatology, The Fourth Military Medical University, Xi’an, Shaanxi 710032 China; 4 State Key Laboratory of Oral & Maxillofacial Reconstruction and Regeneration, National Clinical Research Center for Oral Diseases, Shaanxi Key Laboratory of Stomatology, Department of Operative Dentistry and Endodontics, School of Stomatology, The Fourth Military Medical University, Xi’an, Shaanxi 710032 China

**Keywords:** Nanotopography, SOCE, Cell migration, Live-cell imaging, Opaque surface, Stim1

## Abstract

The exploration of cell response to nanotopography has attracted considerable attentions for years. This article focuses on the influence of nanotopography on the intracellular Ca^2+^ dynamics, the most ubiquitous but ignored second messenger. The classic titanium nanotubes (NT) were fabricated by anodization to formulate nanoporous surfaces. Firstly, the store operative calcium entry (SOCE) in endoplasmic reticulum (ER) and functional Ca^2+^ release-activated Ca^2+^ (CRAC) channels were significantly enhanced on NT surfaces that revealed by live-cell Ca^2+^ imaging and fluorescence resonance energy transfer (FRET) identification of orai1-stim1 connection. To investigate the potential implication of Ca^2+^ elevation, the dynamic cell migration trajectory was monitored by a self-made holder, which could not only be suitable for the opaque implant surface but also guarantee the focus fields identical during samples shifting. The cell migration on NT surface was more vigorous and rapid, which was correlated with higher focal adhesion proteins expression, Ca^2+^-dependent calpain activity and stim1 level. In conclusion, this study has confirmed the novel ER Ca^2+^ hemostasis pathway on nanosurfaces and its crucial role in cell migration regulation, which may help for more biofavorable implant surface design.

**Graphical Abstract:**

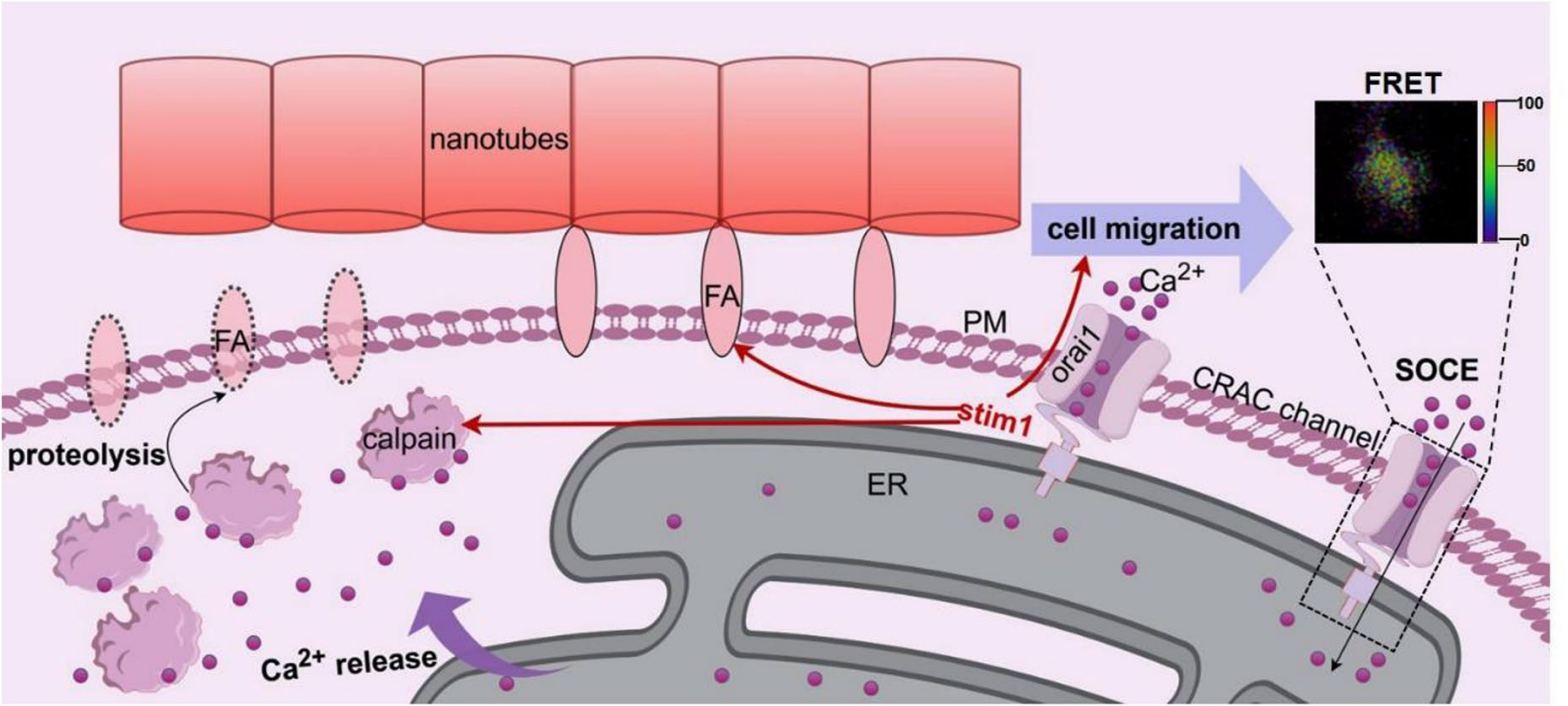

**Supplementary information:**

The online version contains supplementary material available at 10.1186/s12951-023-02249-8.

## Introduction

The cell response to nanotopography has attracted considerable attentions for years [1, 2]. It is not only a bridge between nanotechnology and biological behaviors, but also contribute to the biomedical devices surface design. In the orthopedic implant area, the implant surface nanotopography is known to be prominent for guiding various cell types that contributing to the final osseointegration [3, 4]. For instance, the bone mesenchymal stem cells (BMSCs) are prone to osteogenic differentiation in contact with mild chaotic nanotopography [5]. The macrophages polarization is also directed by nanotopography and contributing to osteogenesis through cytokines secretion, which is also known as the osteoimmunology [6]. In the meanwhile, the mechanism of how the extracellular physical pattern signaling is transferred into cells is also fascinating. Generally, there are several different directions for the explanations. A common direction is mechanical force driven cell deformation. The different nanotopographies may be sensed as different mechanical force that resulting in the alterations of membrane rigidity [7], cytoskeleton arrangement [8, 9] and nucleus deformation [10]. For example, nanoscale surface topography reduces cell stiffness by enhancing integrin endocytosis [7]; Silicon nanoneedle affects nuclear envelope integrity [10]. The responses of cells are critically influenced by different diameters or dimensions of the nanostructure, which is also the outcome of mechanical force driven cell deformation. Adhesion, proliferation, migration, and differentiation of mesenchymal stem cells (MSCs) are maximally induced on 15-nm nanotubes, but prevented on 100-nm nanotubes, which induces cell death [11]. The hydrophilicity is also involved, which contributes to the clinical success of dental implant [12–14]. For instance, cells on hydrophilic modified sand-blasted acid etched titanium surfaces display enhanced adhesion and spreading, as well as increased proliferation [12]. A hydrophobic characteristic has been shown to be a more general characteristic of microstructured titanium [15, 16], while the existence of nanostructures was proven on hydrophilic blasted/etched implant surfaces [15]. Some studies focus on conventional molecular biological regulations feedback, such as Wnt signaling [17], and Mitogen-activated protein kinase (MAPK) pathway [18]. Certain extracellular proteins adsorption on different nanotpographies may act as different signaling transduction cascade to initiate the downstream molecular reactions. Previously, our group has also revealed the mechanotransduction pathway mainly from this direction in various cell types [19–21].

Compared to biomolecules, ions are more extensively distributed in vivo. However, the ions mediated cell behavior has not arisen sufficient concentration. As a second messenger, calcium ions (Ca^2+^) impact nearly all aspects of cellular physiology [22]. Various different Ca^2+^ channels finely maintain and control the Ca^2+^ concentrations at different places to regulate specific cellular activities. Our previous study has confirmed the store operated Ca^2+^ entry (SOCE) in endoplasmic reticulum (ER) has crucial role for osteogenic differentiation control by titania nanotubes array [23]. Depletion of ER Ca^2+^ into cytosol through inositol 1,4,5-trisphosphate receptor (IP3R) is the most efficient way to increase cytosolic Ca^2+^ level for triggering the following Ca^2+^ dependent molecules interaction [24]. Apart from cell differentiation, the SOCE process is mostly validated for cell migration regulation [25, 26], which is a prerequisite step post cell adhesion on biomaterial surface. In the meanwhile, the ions oscillations are often transient at minutes scale and dependent on living status. Limited by normal imaging techniques, it is difficult to monitor ions alterations in living cells on untransparent biomaterial surface, such as the metallic implant surface.

In the present study, we have fabricated two devices for living cells imaging on untransparent biomaterial surface by confocal microscopy. The cytosolic Ca^2+^ oscillations and SOCE process were successfully captured on titanium implant surface with different nanotopographies. Meanwhile, their relevance to cell migration was also monitored through live cell trajectory mapping. This work not only for the first time directly revealed the Ca^2+^ alterations and endoplasmic reticulum and plasma membrane (ER-PM) contact site that interpreted by nanotopography, but also implied a new unfiled but important area for the investigation of cell responses to biomaterial surface.

## Experimental section

### Titanium (Ti) samples Preparation and characterization

Pure Ti plates (99.997% purity, diameter = 15 mm, thickness = 1 mm) were provided by Baoji Nonferrous Metals Industry Corporation (Baoji, China). Then, the samples were polished from 400 to 7000 meshes with SiC sandpaper and ultrasonically cleaned sequentially in acetone, absolute ethanol, and deionized water to produce the polished Ti (PT) surface. The nanotube topography (NT) was obtained on PT surface via anodization in 0.5 wt% hydrofluoric acid at either 5 V (NT5) or 20 V (NT20) for 1 h [6, 27]. The field-emission scanning electron microscope (FE-SEM; Hitachi Ltd, Japan) was used to observe the surface morphology. All samples were sterilized with 75% ethanol prior to cell culture.

### Cell culture

The primary murine bone marrow mesenchymal stem cells (mBMSCs) were used in this study and all the animal experimental protocols were approved by the Animal Ethics Committees of the Fourth Military Medical University. Briefly, the femurs and tibias of male C57BL/6 mice aged 6–8 weeks were isolated. The bone marrow contents were flushed out from the diaphysis with Minimum Essential Medium Alpha basic (α-MEM, Gibco, USA) supplemented with 20% fetal bovine serum (FBS, InCellGenE, USA), and 1% penicillin-streptomycin (HyClone, USA), and filtered by cell strainer of 100 μm. After 4 days culture, half of the medium was refreshed, and after 6 days, all the medium was refreshed. When the cells reached to ~ 80% confluence, the mBMSCs were trypsinized using 0.25% typsin (Gibco) for passage culture. The passage 2 and 3 of cells were used in the experiments.

### Cytosolic Ca^2+^ measurement

For Ca^2+^ imaging, cells on Ti samples were incubated with 10µM FLUO-8 AM (Abcam, UK) for 30 min. Then the cells were imaged by confocal laser scanning microscope (Olympus, Japan) in Ca^2+^ -free medium containing 140 mM NaCl, 5 mM KCl, 1 mM MgCl_2_, 10 mM glucose (all from the Sinopharm Chemical Reagent Co., Ltd, China), 10 mM HEPES, and 20 µM EGTA (both from Sigma, USA), with or without Ca^2+^ release-activated Ca^2+^ (CRAC) inhibitor CM4620 (10 µM, MCE, USA). An ultra-thin Ti cross (side length 19 mm, thickness 0.45 mm) was used to support the samples in order to allow fluids exchange in the gap between glass bottom and living cells. The observation time-frame was 10 min for each sample, consisting of 1 min adaption, 4 min Thapsigargin (TG, 2.5 µM, Invitrogen, USA) treatment, and 5 min Ca^2+^ (2 mM, Sinopharm Chemical Reagent Co., Ltd) recovery.

### Transmission electron microscopy (TEM) examination

After cultured for 24 h, 7 × 10^6^ cells on the surface of each group were collected using 0.25% trypsin (Sigma) and fixed in 2.5% glutaraldehyde, postfixed with 1% osmium tetroxide, dehydrated in ethanol, and embedded in Epoxy. After sectioning, the intracellular ER and plasma membrane (PM) structure were observed in a transmission electron microscope (FEI, USA).

### Fluorescence resonance energy transfer (FRET) measurement

FRET was performed on confocal laser scanning microscope (Nikon Instruments Inc., USA). For GFP and mCherry excitation, 445 and 514 nm lasers were chosen respectively. GFP and mCherry fluorescent signals were collected at 465–505 nm and 518–558 nm separately. Throughout the data acquisition process, samples were maintained at room temperature.

For FRET measurement, mBMSCs were infected by lentiviruses of only orai1-GFP, only stim1-mCherry, and both orai1-GFP and stim1-mCherry respectively. After 3 days, the cells were collected using 0.25% trypsin (Sigma) and seeded on different surfaces. After 24 h, the samples were inverted on the titanium cross in the confocal dishes containing Ca^2+^-free medium. The observation time-frame was 9 min for each sample, consisting of 1 min adaption, 4 min TG (2.5 µM, Invitrogen) treatment, and 4 min Ca^2+^ (2 mM, Sinopharm Chemical Reagent Co., Ltd) recovery. Time-lapse images were captured every 30s for a 9 min period. The final datasets were analyzed using the FRET module in the Nikon NIS-Elements software, and calibrated according a previously published method [28]. At last, the average FRET efficiency was obtained from the FRET module and used for statistical analysis.

### Cell trajectory tracking

The cells were seeded on different surfaces for 24 h. Next, the cell plasma membrane was stained by Cell Plasma Membrane Staining Kit with DIO (Green fluorescence) (Beyotime, China) according to the instructions. Then the specimens were mounted in a novel self-made holder, which was precisely suitable for 24-Well Glass Bottom Black Plate with Lid (Cellvis, USA). The cells were cultivated in a live-cell incubation chamber mounted on a live-cell workstation (Tokai Hit, Japan). The temperature and atmosphere conditions inside the chamber were controlled to be the same as the cell incubator. Live-cell time-lapse imaging was performed on confocal laser scanning microscope (Nikon Instruments Inc.). Cell images were captured every 15 min for a total duration of 12 h. To quantify cell migration, cell images were imported into the ImageJ software, and the manual tracking function in ImageJ software was used to obtain the trajectory of cell, including the coordinates and length of the trajectory. Then, the position plots of the cell sites were mapped to a common origin using MATLAB software.

### Immunofluorescence staining

The visualization of the desired focal adhesion (FA) proteins including Vinculin and FAK was carried out by immunofluorescent staining. The cells were seeded on different surfaces at the density of 1 × 10^5^ cells per sample and cultured for 24 h. Then the cells were gently rinsed with phosphate-buffered saline (PBS; HyClone, USA) and fixed with 4% paraformaldehyde. After permeabilized with 0.1% Triton X-100 for 10 min, the cells were blocked with 5% goat serum albumin (Boster, USA) for 30 min. Then the cells were incubated with anti-vinculin antibody (1:100, Abcam) or anti-FAK antibody (1:400, Proteintech, USA) at 4 ℃ overnight. The cells were labeled with Dylight 488, goat anti-rabbit IgG second antibody (1: 200, Abbkine, USA) for 2 h at 37 ℃. Finally, the cells were mounted by Antifade Mounting Medium with DAPI (Beyotime) and imaged using confocal laser scanning microscope (Nikon Instruments Inc.). The fluorescent intensity of the cells was analyzed using the ImageJ software.

### Calpain activity

The cells were seeded on different surfaces at the density of 2 × 10^5^ cells per sample and cultured for 24 h. After rinsed with PBS, the cells were incubated with 10 µM CMAC peptidase substrate, t-BOC-Leu-Met (Invitrogen) at 37 ℃ for 30 min. The blue fluorescence of t-BOC-Leu-Met cleavage products by calpain was dynamically examined in living cells under Ca^2+^ -free condition by confocal at Ex/Em of ~ 351/430 nm.

### Statistical analysis

All data were expressed as mean ± SD, and at least three independent experiments for each group were performed. The statistical analysis was conducted using GraphPad Prism 8.0 software. The results were analyzed by one-way ANOVA and Tukey’s post hoc tests. A *p* value less than 0.05 is statistically significant.

## Results and discussion

### CRAC channels participate in SOCE on Ti surfaces and SOCE level is up-regulated by NT

**Fig. 1:**
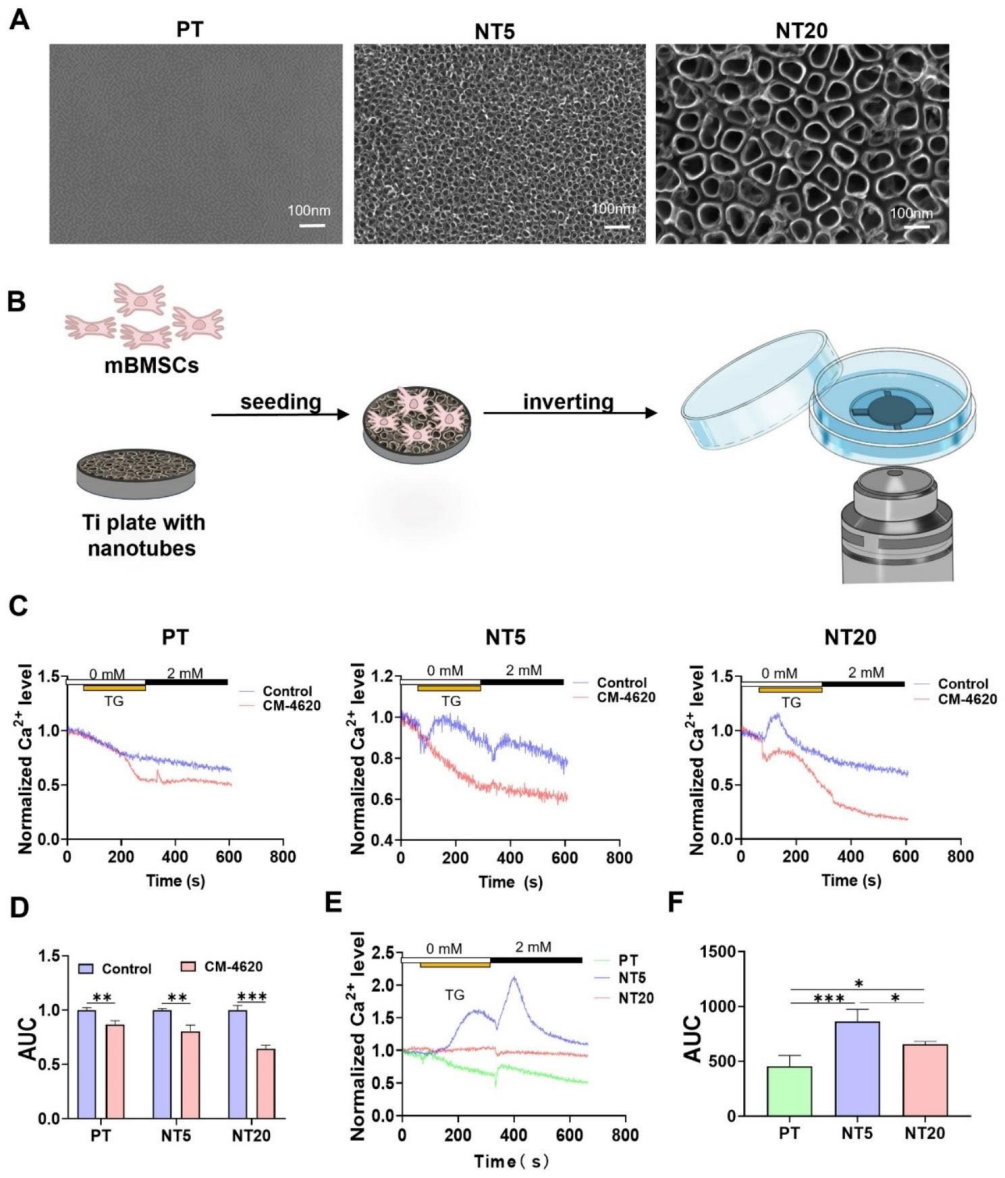
Observation the SOCE on different Ti surfaces via Ti cross which was used to assist in observing living cells on the surface of opaque materials. (**A**) FE-SEM images of the sample surfaces. (**B**) Schematic diagram of observing calcium ion oscillation in living cells on the surface of titanium samples using laser confocal microscope and assisted by Ti cross. (**C**) Calcium traces of the cells on Ti plate treated with CM-4620, when the fluorescence of Calcium was monitored, the 2.5 µM TG was added, following by 2mM Calcium. (**D**) The quantification of the area under the curve (AUC) (n = 3). (**E**, **F**) Calcium fluxes and AUC following stimulation with TG and 2 mM calcium in mBMSCs on different surfaces (n = 4). Data are presented as mean ± SEM, Student’s t-test or One-way ANOVA, *P ≤ 0.05, **P ≤ 0.01, ***P ≤ 0.001

Uniform TiO_2_ tubular nanostructure could be observed on both the surfaces of NT5 and NT20, and the inner diameters of the nanotubes were about 25 and 80 nm respectively (Fig. 1A). The NT surface also showed hydrophilic property with increased roughness (Fig. S1), which was in line with our previous sample surfaces. In order to realize in situ observation of living cells on opaque Ti surfaces, a specially designed Ti cross was applied. The Ti cross with 0.45 mm thickness was used to support the inverted Ti plate seeded with cells, which could ensure the cells to be in the confocal focusing range without any pressure and the quick spreading of the drug added to the confocal dish (Figs. 1B and S3). To verify if TG induced Ca^2+^ influx on Ti surfaces was through CRAC channels, a CRAC channel inhibitor, CM-4620 was applied. CM-4620 showed inhibition effects on Ca^2+^ influx both in ER Ca^2+^ depletion stage induced by TG and Ca^2+^ recovery stage in all the three surfaces (Fig. 1C and D). Next, the impact of NT on cytosolic Ca^2+^ level was investigated. Ca^2+^ level was significantly higher in NT5 and NT20 compared with PT, and NT5 showed the highest Ca^2+^ level (Fig. 1E and F).

The rise in cytosolic Ca^2+^ level caused by NT may come from two ways, entry across the plasma membrane or release from intracellular stores [29]. The cells on nanotubes are forced to elongate and stretch to search for protein aggregates, thus exposed to more stress and changes in membrane tension compared to PT [30]. So the mechanosensitive ion channels may altered in response to changes in membrane tension and curvature [31], leading to elevated intracellular calcium levels [32]. ER is recognized as an important intracellular Ca^2+^ store [33]. Meanwhile, IP_3_R-mediated ER Ca^2+^ release participates in overall intracellular Ca^2+^ signaling network and regulates fundamental cellular function [24]. SOCE, activated by reduction of free [Ca^2+^]_ER_, has been reported to play an important role in many physiological activities, such as spatiotemporal control of gene expression, maintenance of immune function, and cell migration [34]. So, the above experimental results indicates that the ER Ca^2+^ release may play critical physiological role, which implies the importance of further analyzing SOCE in ER.

As the constituent molecules of CRAC channels, orai proteins are identified as store-operated Ca^2+^ channels and stim proteins are identified as Ca^2+^ sensors for SOCE [34]. There are three homologues for orai, the orai1/2/3, and two homologues for stim, the stim1/2. Although orai1 and stim1 are reported to be the key components of CRAC channel [35], orai2/3 and stim2 also contribute to SOCE through different ways [36, 37]. Therefore, which subtypes dominated on NT surface should be confirmed.

Polymerase chain reaction (PCR) examination of the dynamic mRNA levels, orai1 and Stim1 were most sensitive to time compared with orai2/3 and stim2 in NT5 and NT20, while there were no significant differences in the expression of orai1/2/3 and the expression of stim1/2 at different times in PT (Fig. S4A and B). Thus, orai1 and stim1 were selected as the target proteins for following observation. Compared with 3 and 7 days, at 1 day, the difference of orai1 or stim1 among PT, NT5 and NT20 was the greatest (Fig. S4C). Therefore, 1 day was selected as the target time point for subsequent observation. Meanwhile, NT had promoted the expression of orai1 and stim1 compared with PT, and the expression of stim1 in NT5 was higher than that in NT20 (Fig. S4C), this could also partially explain the elevated cytosolic Ca^2+^ in NT5 and NT20, especially the NT5.

### NT enhances FRET efficiency between Orai1 and Stim1

**Fig. 2:**
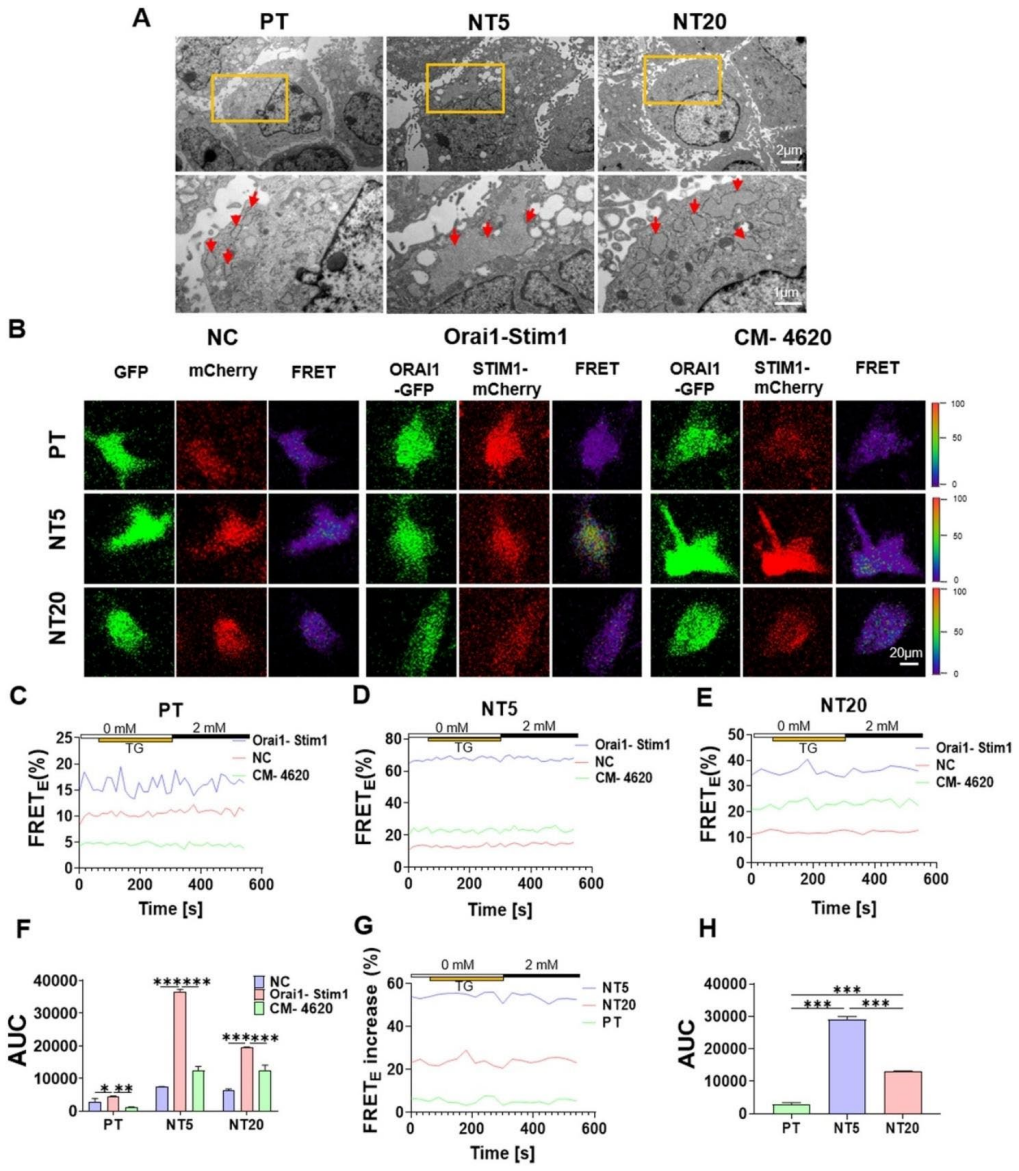
Influence of Ti nanotopographies on the endoplasmic reticulum and FRET between Orai1 and Stim1 in mBMSCs. (**A**) ER and PM observation in mBMSCs on different surfaces by TEM. (**B**) Presentative images of FRET efficiency between orai1-GFP and stim1-mCherry with or without CM-4620 on surfaces of PT, NT5, NT20 at 0 s. (**C-E**) FRET efficiency analysis of the time course of interactions between orai1-GFP and stim1-mCherry with or without CM-4620 on surfaces of PT, NT5, NT20, when the fluorescence was monitored, the 2.5 µM TG was added at 60 s, following by 2mM Calcium at 300s, (**F**) and the quantification of the area under the curve (AUC). (**G**) FRET efficiency increase of the time course of orai1-stim1 group compared with NC group between different surfaces (**H**) and the quantification of the AUC. Data are presented as mean ± SEM, n = 3, Student’s t-test or One-way ANOVA, ns, not significant, *P ≤ 0.05, **P ≤ 0.01, ***P ≤ 0.001

Orai1 is expressed in plasma membrane and stim1 is expressed in the ER membrane. After depletion of ER Ca^2+^, the stim1 and orai1 redistribute within their respective membrane and coaccumulate in clusters to activate SOCE [38]. The sites of coaccumulation are ER-PM junctions, where ER is held close enough but not fused to the PM, and the average ER-PM gap is reported to range from 10 to 17 nm [39]. So the observation of ER-PM junctions can partially reflect the relative positional relation between orai1 and stim1. ERs were observed to be more swollen in NT5 and NT20 than PT, and NT5 was more pronounced than NT20 (Fig. 2A). And this result is agreed with our previous research that ER could suffer from different stress states triggered by morphological factors [23, 40]. Then ER stress leads to Ca^2+^ releasing from ER to cytosol [41], which is also responsible for the high Ca^2+^ cytosolic level in NTs, especially the NT5. However, no typical ER-PM junctions were observed in both NT5, NT20 and PT using TEM (Fig. 2A), which may be due to the digestion procedure. Thus, the in-situ FRET observation was performed.

As a highly effective means of studying protein interactions [42], FRET was used to explore the positional relation between orai1 and stim1 based on in situ live-cell observation. FRET occurs in a short range (5-10 nm) across which energy is transferred from an excited donor to acceptor fluorophore [42]. Firstly, FRET phenomenon was verified emerging in both PT, NT5 and NT20, and occurring between orai1 and stim1 by adding CRAC inhibitor, the CM4620 (Fig. 2B-F). NT could promote the FRET efficiency between orai1 and stim1 compared with PT, especially the NT5 (Fig. 2B, G, H). Comparing with PT, the stronger expression of stim1 in NT5 and NT20 (Fig. S4C), especially in NT5, can partially explain the higher FRET efficiency in NT5 and NT20, especially in NT5. However, with the addition of TG and Ca^2+^, no evident fluctuation of FRET efficiency was occurred (Fig. 2G).

It is speculated that in order to maintain the basic functions of cells, Ca^2+^ is occasionally discharged from ER into cytoplasm, and the Ca^2+^ restoration in ER is replenished through CRACs. Nevertheless, due to the Ca^2+^ free medium used in this experiment, no Ca^2+^ could be replenished to ER. So it is speculated that the low ER Ca^2+^ will activate all functional CRAC channels, which resulting in no increase in FRET efficiency even after TG addition. Even the addition of Ca^2+^ can’t induce any change in FRET efficiency, as SOCE may be not ending in 5 min [34], thus the CRAC channels are keeping activated.

### Cell Migration speed and length are promoted by NT surface through Stim1

Previous studies have confirmed the alterations of ER SOCE and specific orai1-stim1 approaching pattern induced by NT surface, it is reasonable to elucidate their biological implications. Accordingly, Ca^2+^ signaling is vastly related to cell adhesion and migration, in which stim1 expression is directly associated with tumor cells invasiveness and embedding [43]. In the meanwhile, the cell migration is also a perquisite process post cell contact with implant surface. Therefore, the role of stim1 in the cell migration regulation on NT surface was investigated.

**Fig. 3:**
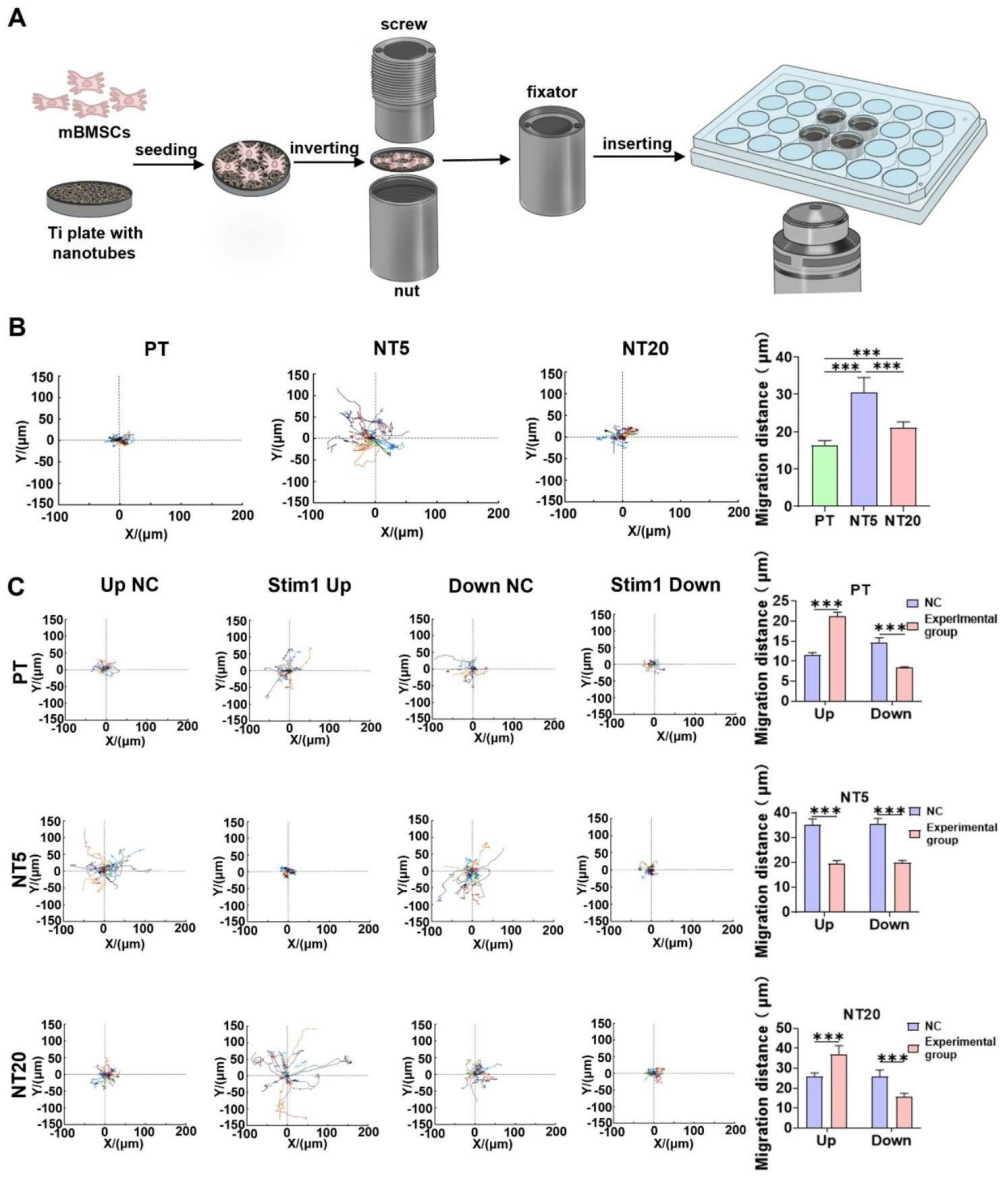
Influence of Ti nanotopographies and stim1 on the migration of single cell. (**A**) Schematic diagram of observing single cell migration on the surface of titanium samples using laser confocal microscope and assisted by self-made holder. (**B**) The single cell migration trajectory on different surfaces, and position plots of the cell sites mapped to a common origin, which showed fifteen typical cells in each group. Each site represents the time-dependent position of the center of the nucleus of a single cell. And the quantification of the cell migration distance on different surfaces (n = 22). (**C**) The single cell migration trajectory after knockdown or overexpression of stim1 on three surfaces and the quantification of the cell migration distance (n = 15). Data are presented as mean ± SEM, Student’s t-test or One-way ANOVA, ns, not significant, *P ≤ 0.05, **P ≤ 0.01, ***P ≤ 0.001

Collective cell migration experiments were tried firstly to explore the motility of mBMSCs. The mobility of mBMSCs on NT5 and NT20 surfaces was obviously increased compared to PT surface (Fig. S6). In order to realize in situ observation of living cells migration on opaque materials, a specially designed holder was applied (Figs. 3A and S8). The holder could keep the Ti samples stable during the rapid movement of plate, which guaranteed the multiple focused areas unchanged. mBMSCs exhibited the most vigorous motility on NT5 surface, followed by NT20, and the last was PT, as displayed in the trajectory analysis obtained from the time-lapse imaging (Figs. 3B and S9A). In addition, the cell migration was increased or decreased after stim1 overexpression or inhibition, except for NT5 that the migration was not increased anymore after stim1 overexpression (Figs. 3C, S9B, C).

It is consistent with previous reports that NT promoted cell migration [44–46]. It has been verified that cell migration is induced by dynamically regulated of the assembly and disassembly of FA proteins [47]. Meanwhile calcium influx has been linked to FA turnover during cell migration [48]. Therefore, it can be speculated that different calcium levels in NT20, NT5 and PT may lead to different FA dynamics, resulting in different migration behaviors mentioned above. However, there is a limit for the influence of Ca^2+^, so the cell migration in NT5 doesn’t continue to increase after overexpression of stim1.

### Focal Adhesion Proteins are upregulated on NT surface and regulated by Stim1

**Fig. 4:**
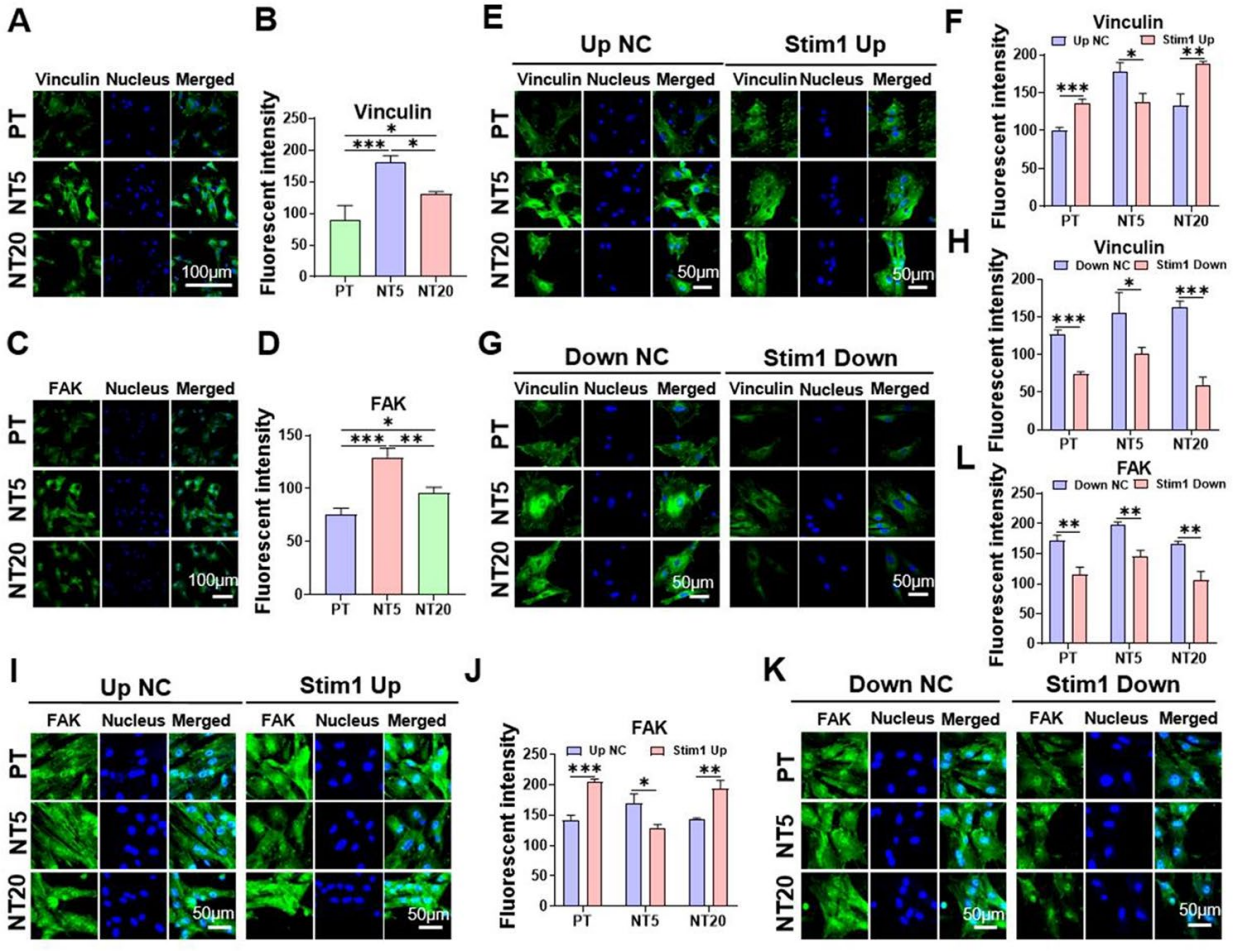
Influence of Ti nanotopographies and stim1 on FAK and Vinculin of mBMSCs. (**A**) Immunofluorescence staining depicts the expression of vinculin on different surfaces (**B**) and the quantification of fluorescence intensity of vinculin. (**C**) Immunofluorescence staining depicts the expression of FAK on different surfaces (**D**) and the quantification of fluorescence intensity of FAK. (**E-H**) The expression of vinculin on different surfaces after overexpression (**E**) or knockdown (**G**) of stim1 exhibited by immunofluorescence staining, and the quantification of fluorescence intensity of vinculin (**F**,**H**). (**I-L**) The expression of FAK on different surfaces after overexpression (**I**) or knockdown (**K**) of stim1 exhibited by immunofluorescence staining, and the quantification of fluorescence intensity of vinculin (**J,L**). Data are presented as mean ± SEM, n = 3, Student’s t-test or One-way ANOVA, *P ≤ 0.05, **P ≤ 0.01, ***P ≤ 0.001

The focal adhesion proteins of vinculin and FAK were observed by immunofluorescent staining. Comparing with PT, the expressions of vinculin and FAK were promoted by NT5 and NT20, with NT5 having the strongest expression (Fig. 4A-D). They were also upregulated after stim1 overexpression, except for NT5 (Fig. 4E, F, I, J). Meanwhile, the expressions of vinculin and FAK in all three groups were inhibited after stim1 knock-down (Fig. 4G, H, K, L).

For poorer hydrophilicity was exhibited in PT than NT5 and NT20 (Fig. S1A), there may be lower population of serum proteins aggregating on the surface of PT [30], which is adverse to the formation of FA. The stronger expression of FAs in NT5 compared to NT20 may be explained by two reasons. On the one hand, the protein aggregates initially attach only to the available surfaces that are the top portion of the nanotube walls [30], and the adhesion of proteins (≈ 30-nm-size regime) initially determines the degree of cell adhesion. The nanopores with a diameter of 25 nm (NT5) are suitable for ≈ 30-nm size serum protein aggregates to anchor on, while the nanopores with a diameter of 80 nm (NT20) are too large for the serum proteins to aggregates to anchor on. On the other hand, since lateral spacing of integrin ligands influencing FA assembly [49], and the lateral spacing of integrin ligands less than 50 ~ 70 is essential for the assembly of FA and stress fibers [50], therefore 80-nm diameter nanopores (NT20) will inhibit the assembly of FA compared to 25-nm diameter nanopores (NT5). It is showed in studies that the ER protein colocalizes with adhesion sites, and the transportation of ER tubules along microtubules to contact FAs is indeed crucial to support FA growth [51, 52]. Therefore, it is speculated that there were more colocalizations of ER protein with FA, which means more ER distributing near the plasma membrane. This may be responsible for the highest FRET efficiency between orai1 and stim1 in NT5.

Since the calpain family of protease has been reported to regulate cell motility by degrading FA and promoting rear detachment [53], the NT may affect the activity of calpain. If the assumption comes into existence, similarly, is effect of NT on cell migration ultimately achieved through the regulation of calpain activity by stim1?

### Calpain activity is increased on NT surface and regulated by Stim1

**Fig. 5:**
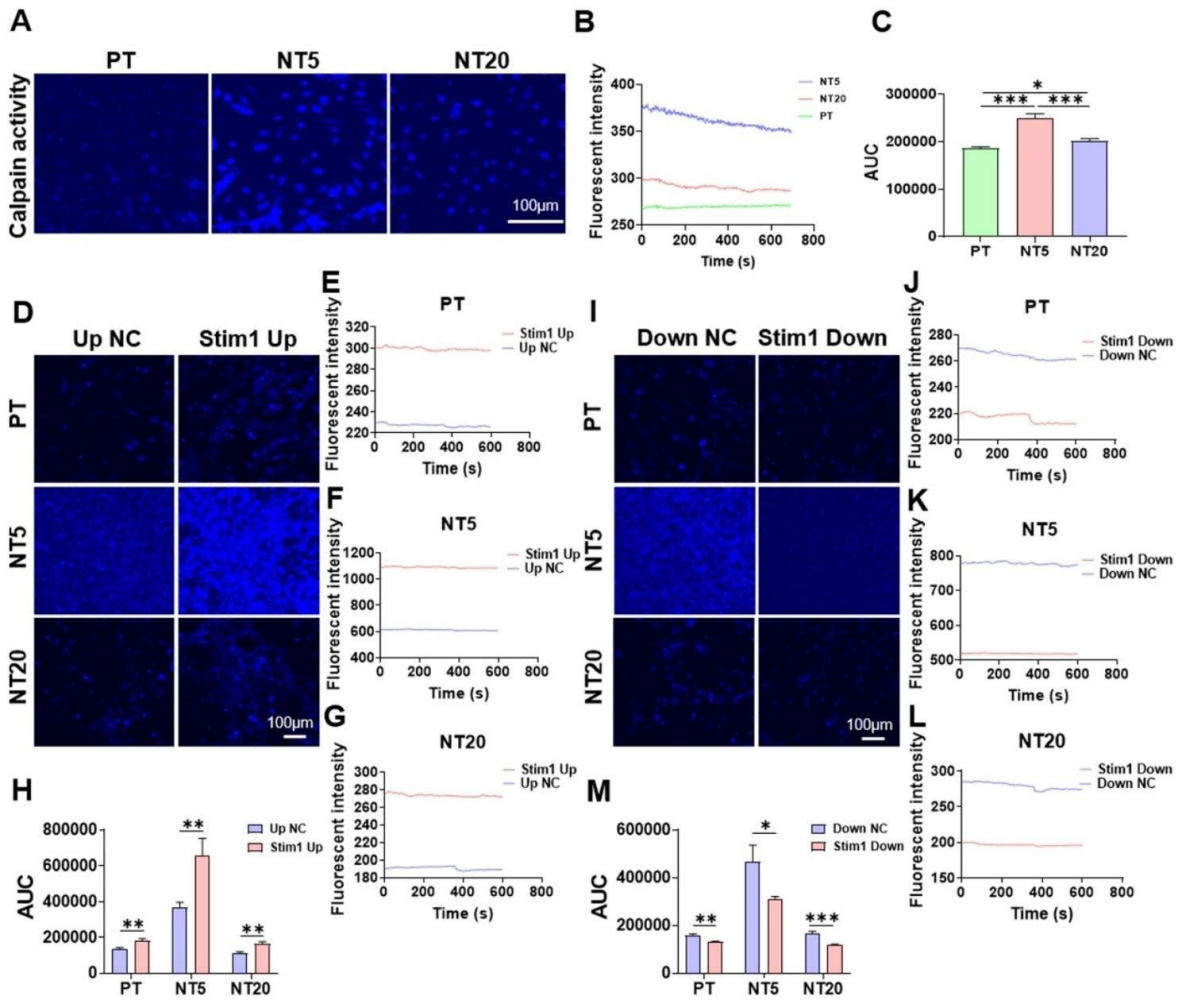
Influence of Ti nanotopographies and stim1 on the activity of calpain of mBMSCs. (**A**) mBMSCs on different surfaces were treated with t-BOC-Leu-Met, which is an artificial fluorogenic calpain substrate, for 30 min. And each sample were observed for 10 min, the confocal images at 0 s were exhibited. (**B**) The time course of fluorescent intensity which represents the time course of calpain activity of mBMSCs on different surfaces. (**C**) and the quantification of the area under the curve (AUC). (**D**) mBMSCs overexpressing stim1 on different surfaces were treated with t-BOC-Leu-Met, and each sample were observed for 10 min, the confocal images at 0 s were exhibited. (**E-G**) The time course of fluorescent intensity of mBMSCs overexpressing stim1 on different surfaces, (**H**) and the quantification of the AUC. (**I**) mBMSCs with stim1 knockdown on different surfaces were treated with t-BOC-Leu-Met, and each sample were observed for 10 min, the confocal images at 0 s were exhibited. (**J-L**) The time course of fluorescent intensity of mBMSCs with stim1 knockdown on different surfaces, (**M**) and the quantification of the area under the curve (AUC). Data are presented as mean ± SEM, Student’s t-test or One-way ANOVA, *P ≤ 0.05, **P ≤ 0.01, ***P ≤ 0.001

The t-BOC-Leu-Met was used to measure the calpain activity in live-cells on different surfaces of Ti samples, with the assistance of Ti cross mentioned above. It was found that NT5 and NT20, especially NT5 can largely promote the activity of calpain compared to PT (Fig. 5A-C). The overexpressed stim1 could promote the activity of calpain in all three groups (Fig. 5D-H). The activity of calpain was decreased by knocking down stim1 in all three groups (Fig. 5I-M).

As calpain exhibits calcium-dependent protease activity, the high level of calcium in NT5 and NT20, especially NT5, can explain the promotion of NT5 and NT20, especially NT5, on the activity of calpain. For overexpressed stim1 may improve SOCE [26], this may explain the high activity of calpain in all three groups. But the hyperactivity of calpain after stim1 being overexpressed in NT5 may result weak FAs in NT5, which is likely to lead to less stable protrusions, thus explains the decrease motility of cells in NT5 [54].

## Conclusion

The study lively revealed the Ca^2+^ oscillation driven by ER SOCE process on opaque implant surface, and its significance on cell migration regulation. In brief, the ER SOCE process was enhanced on nanoporous surface, which accounted for the vigorous cell migration through stim1 upregulation. The findings may not only help to better understand the Ca^2+^ homeostasis induced by nanotopographies, but also imply a method for acquiring concrete living cell information on opaque biomaterial surface. As a classic titanium nanomaterial, NT5 with a diameter of 25nms can better promote early migration of mBMSCs than NT20 with a diameter of 80nms. This may provide a theoretical basis for promoting early osseointegration of implant, but further validation is needed through in vitro and in vivo experiments.

### Electronic supplementary material

Below is the link to the electronic supplementary material.

**Supplementary Material 1: Fig. S1**. Characterizations of the Ti samples. **P ≤ 0.01, ***P ≤ 0.001. **Fig. S2**. Identification of the multidirectional differentiation potential of MBMSCs, theosteogenic differentiation and adipogenic differentiation. **Fig. S3**. The diagram and physical images of the titanium cross used to assist inconfocal imaging of live-cells. **Fig. S4**. Screening of subtypes of constituent Molecules of functional store-operated CRAC channels that are most sensitive to time on Ti surfaces, and screening of the most sensitive time points for changes in these subtypes. *P ≤ 0.05,**P ≤ 0.01, ***P ≤ 0.001. **Fig. S5**. Determination of the optimal multiplicity of infection (MOI) for lentivirus transfection, Orai1-GFP, Stim1-mCherry. **Fig. S6**. Influence of Ti nanotopographies on collective cell migration at 0 h, 6 h, 12 h,24 h, 48 h. Green-F-actin; Blue-nuclei. **Fig. S7**. Screening of stim1 knockdown within three guarantees and validation of RNA levels after stim1 knockdown and overexpression. ***P ≤ 0.001. **Fig. S8**. The diagram and the physical image of the holder used to assist in confocalimaging of live-cells. **Fig. S9**. The live-cell time-lapse imaging and single-cell migration trajectories ondifferent Ti surfaces.

## Data Availability

The datasets used and/or analyzed during the current study are available from the corresponding author on reasonable request.

## Abbreviations

NT: titanium nanotubes
SOCE: store operative calcium entry
ER: endoplasmic reticulum
CRAC: Ca2 + release-activated Ca2+
FRET: fluorescence resonance energy transfer
BMSCs: bone mesenchymal stem cells
MSCs: mesenchymal stem cells
MAPK: Mitogen-activated protein kinase
Ca2+: calcium ions
IP3R: inositol 1,4,5-trisphosphate receptor
ER-PM: endoplasmic reticulum and plasma membrane
PT: polished Ti
NT: nanotube topography
NT5: anodization in 0.5 wt% hydrofluoric acid at 5 V
NT20: anodization in 0.5 wt% hydrofluoric acid at 20 V
FE-SEM: field-emission scanning electron microscope
mBMSCs: murine bone marrow mesenchymal stem cells
α-MEM: Minimum Essential Medium Alpha basic
FBS: fetal bovine serum
TG: Thapsigargin
PM: plasma membrane
FA: focal adhesion
PBS: phosphate-buffered saline
PCR: Polymerase chain reaction
AUC: area under the curve
