# Correction: Investigation of nanotopography on SOCE mediated cell migration via live-cell imaging on opaque implant surface

**DOI:** 10.1186/s12951-023-02288-1

**Published:** 2024-02-06

**Authors:** Yan Zhang, Kai Li, Guangwen Li, Yazheng Wang, Yide He, Wen Song, Yumei Zhang

**Affiliations:** 1 State Key Laboratory of Oral & Maxillofacial Reconstruction and Regeneration, National Clinical Research Center for Oral Diseases, Shaanxi Key Laboratory of Stomatology, Department of Prosthodontics, School of Stomatology, The Fourth Military Medical University, Xi’an, Shaanxi 710032 China; 2 Department of Stomatology, The 986th Air Force Hospital, Xijing Hospital, The Fourth Military Medical University, Xi’an, Shaanxi 710032 China; 3 State Key Laboratory of Oral & Maxillofacial Reconstruction and Regeneration, National Clinical Research Center for Oral Diseases, Shaanxi International Joint Research Center for Oral Diseases, Department of Periodontology, School of Stomatology, The Fourth Military Medical University, Xi’an, Shaanxi 710032 China; 4 State Key Laboratory of Oral & Maxillofacial Reconstruction and Regeneration, National Clinical Research Center for Oral Diseases, Shaanxi Key Laboratory of Stomatology, Department of Operative Dentistry and Endodontics, School of Stomatology, The Fourth Military Medical University, Xi’an, Shaanxi 710032 China

**Correction: Journal of Nanobiotechnology (2023) 21:471** 10.1186/s12951-023-02249-8

 Due to a typesetting mistake, the title of this article was incorrectly given as 'Investigation of nanotopography on SOCE mediated cell migration via live-cell Imaging' but should have been 'Investigation of nanotopography on SOCE mediated cell migration via live-cell Imaging on opaque implant surface’.

The original article [1] has been revised. The publisher apologises to the authors and readers for the inconvenience caused by this error.
