# Supplementary material for: Investigation of nanotopography on SOCE mediated cell migration via live-cell Imaging on opaque implant surface
Source: J Nanobiotechnology. 2023 Dec 8;21:471. doi: 10.1186/s12951-023-02249-8 (PMC10704632; doi:10.1186/s12951-023-02249-8)
Supplement: Supplementary file 1 — Supplementary Material 1: Fig. S1. Characterizations of the Ti samples. **P ≤ 0.01, ***P ≤ 0.001. Fig. S2. Identification of the multidirectional differentiation potential of MBMSCs, theosteogenic differentiation and adipogenic differentiation. Fig. S3. The diagram and physical images of the titanium cross used to assist inconfocal imaging of live-cells. Fig. S4. Screening of subtypes of constituent Molecules of functional store-operated CRAC channels that are most sensitive to time on Ti surfaces, and screening of the most sensitive time points for changes in these subtypes. *P ≤ 0.05,**P ≤ 0.01, ***P ≤ 0.001. Fig. S5. Determination of the optimal multiplicity of infection (MOI) for lentivirus transfection, Orai1-GFP, Stim1-mCherry. Fig. S6. Influence of Ti nanotopographies on collective cell migration at 0 h, 6 h, 12 h,24 h, 48 h. Green-F-actin; Blue-nuclei. Fig. S7. Screening of stim1 knockdown within three guarantees and validation of RNA levels after stim1 knockdown and overexpression. ***P ≤ 0.001. Fig. S8. The diagram and the physical image of the holder used to assist in confocalimaging of live-cells. Fig. S9. The live-cell time-lapse imaging and single-cell migration trajectories ondifferent Ti surfaces. [file 12951_2023_2249_MOESM1_ESM.docx]

**Investigation of Nanotopography on SOCE Mediated Cell Migration via Live-cell**

**Imaging on Opaque Implant Surface**

Yan Zhang^1, †^, Kai Li^2, †^, Guangwen Li^1, †^, Yazheng Wang^3^, Yide He^4, *^, Wen Song^1, *^, and Yumei Zhang^1, *^

*1 State Key Laboratory of Oral & Maxillofacial Reconstruction and Regeneration, National Clinical Research Center for Oral Diseases, Shaanxi Key Laboratory of Stomatology, Department of Prosthodontics， School of Stomatology, The Fourth Military Medical University, Xi'an 710032, Shaanxi, China*

*2 Department of stomatology, The 986th Air Force Hospital, Xijing Hospital, The Fourth Military Medical University, Xi'an 710032, Shaanxi, China*

*3 State Key Laboratory of Oral & Maxillofacial Reconstruction and Regeneration, National Clinical Research Center for Oral Diseases, Shaanxi International Joint Research Center for Oral Diseases, Department of Periodontology, School of Stomatology, The Fourth Military Medical University, Xi'an 710032, Shaanxi, China*

*4 State Key Laboratory of Oral & Maxillofacial Reconstruction and Regeneration, National Clinical Research Center for Oral Diseases, Shaanxi Key Laboratory of Stomatology, Department of Operative Dentistry and Endodontics, School of Stomatology, The Fourth Military Medical University, Xi'an 710032, Shaanxi, China*

*† Yan Zhang, Kai Li and Guangwen Li contributed equally to this work.*

** Corresponding authors.*

*E-mail:* [*heyide1227@126.com*](mailto:heyide1227@126.com) *(Yide He); wensong71@163.com (Wen Song); wqtzym@fmmu.edu.cn (Yumei Zhang).*


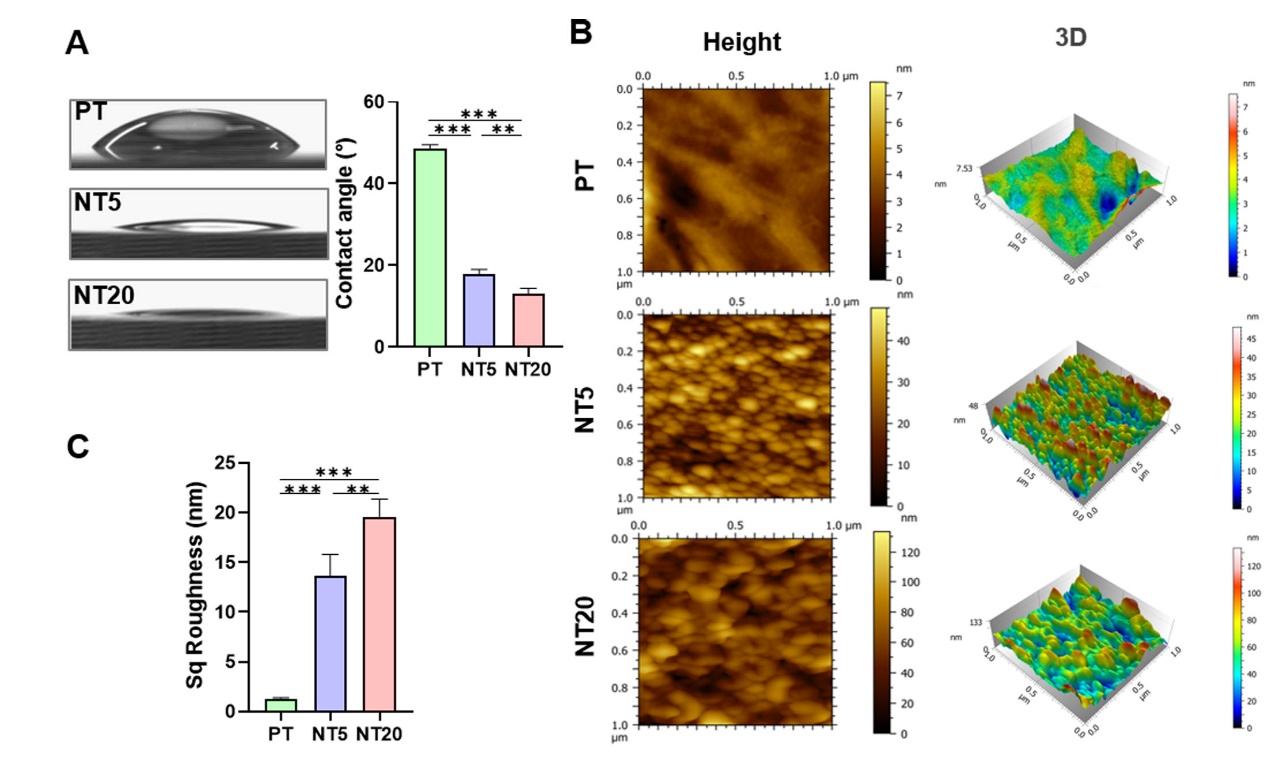


**FigureS1.** Characterizations of the Ti samples. (A) Water contact angle of samples. (B) Top view and 3D of surface height fluctuations of AFM scanning images. (C) Sq roughness of different surfaces. Data are presented as mean ± SEM, n = 3, One-way ANOVA, **P ≤ 0.01, ***P ≤ 0.001.


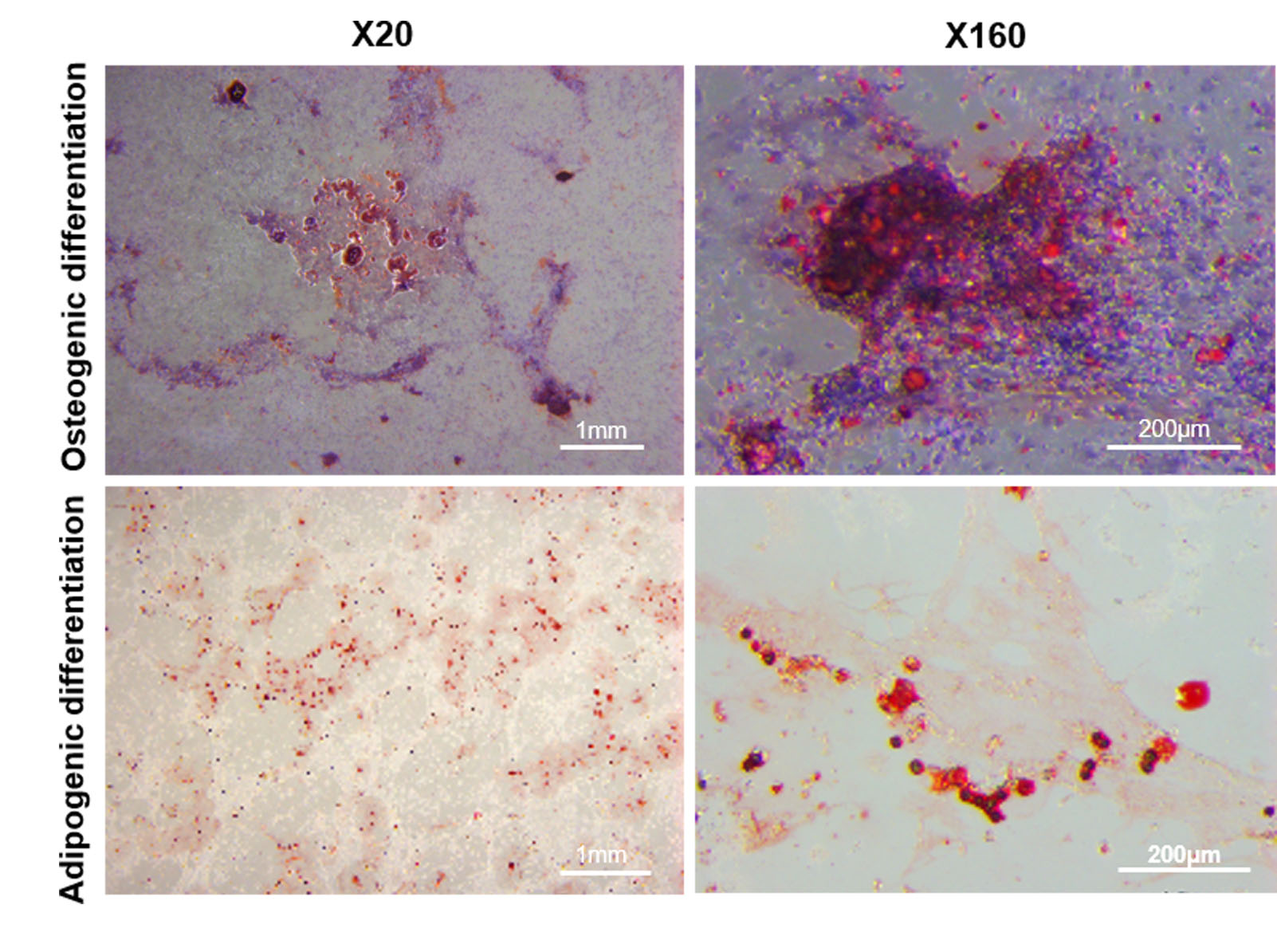


**Figure S2.** Identification of the multidirectional differentiation potential of mBMSCs, the osteogenic differentiation and adipogenic differentiation


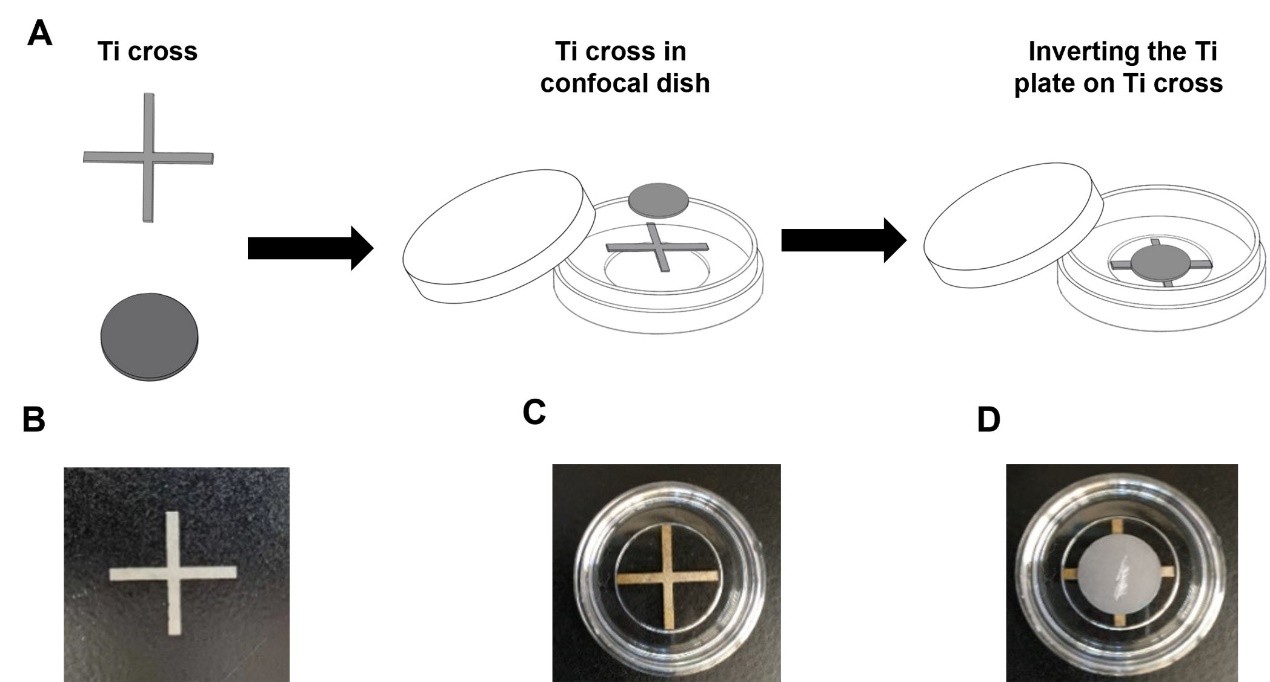


**Figure S3.** The diagram and physical images of the Titanium cross used to assist in confocal imaging of live-cells. (A) The diagram of Ti cross used to support Ti plate in confocal imaging, the Ti plate seeded with live-cells were inverted on the cross. (B) The Ti cross, of which the side length is 19mm, the thickness is 0.45mm. (C) Placing the Ti cross in confocal dish containing Ca^2+^-free medium. (D) The Ti sample seeded with living mBMSCs was inverted on Ti cross.


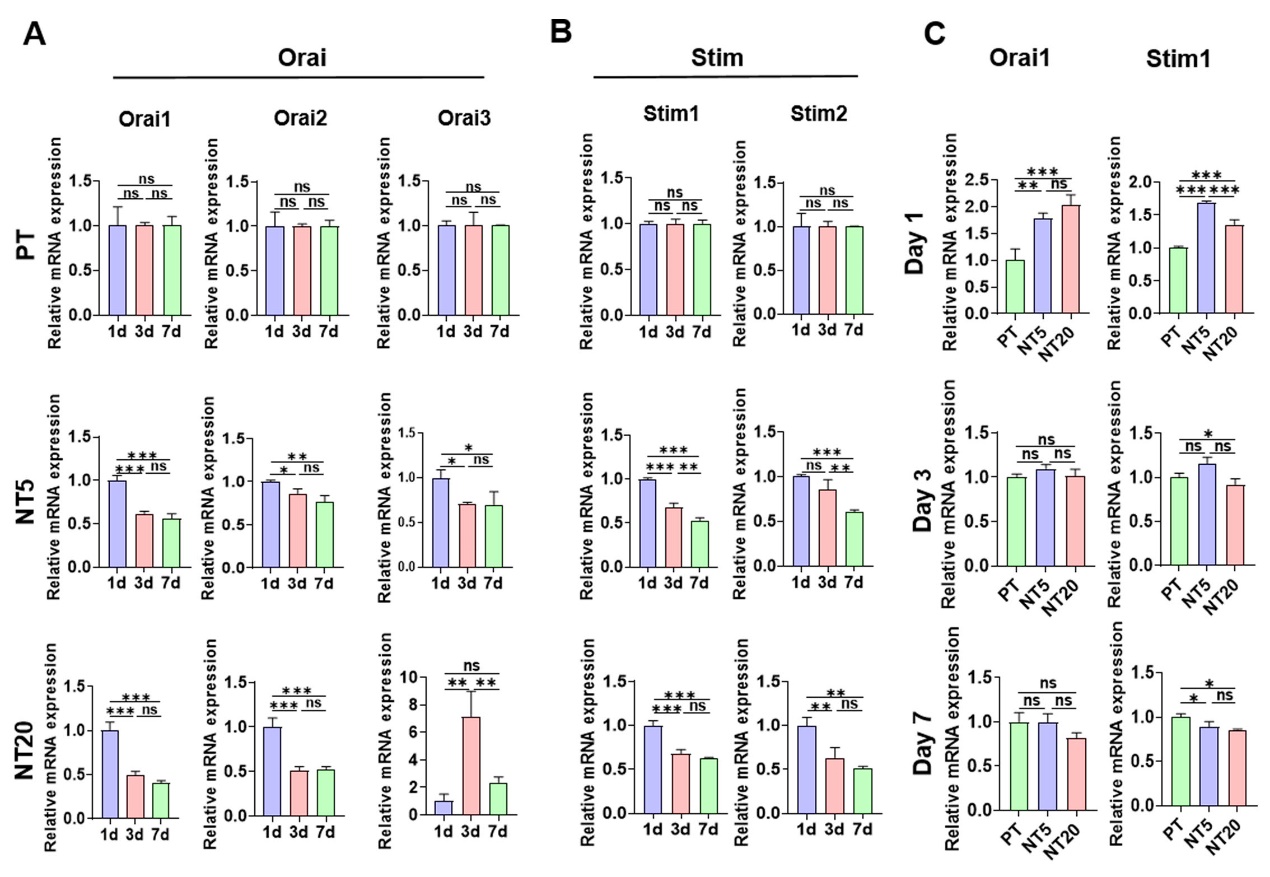


**FigureS4.**Screening of subtypes of constituent Molecules of functional store-operated CRAC channels that are most sensitive to time on Ti surfaces, and screening of the most sensitive time points for changes in these subtypes. (A, B) The orai1/2/3, stim1/2 expression at different time points on three surfaces determined by RT-qPCR. (C) The selected orai1, stim1 expression on different surfaces at three time points determined by RT-qPCR. Data are presented as mean ± SEM, n = 3, One-way ANOVA, ns, not significant, *P ≤ 0.05, **P ≤ 0.01, ***P ≤ 0.001.


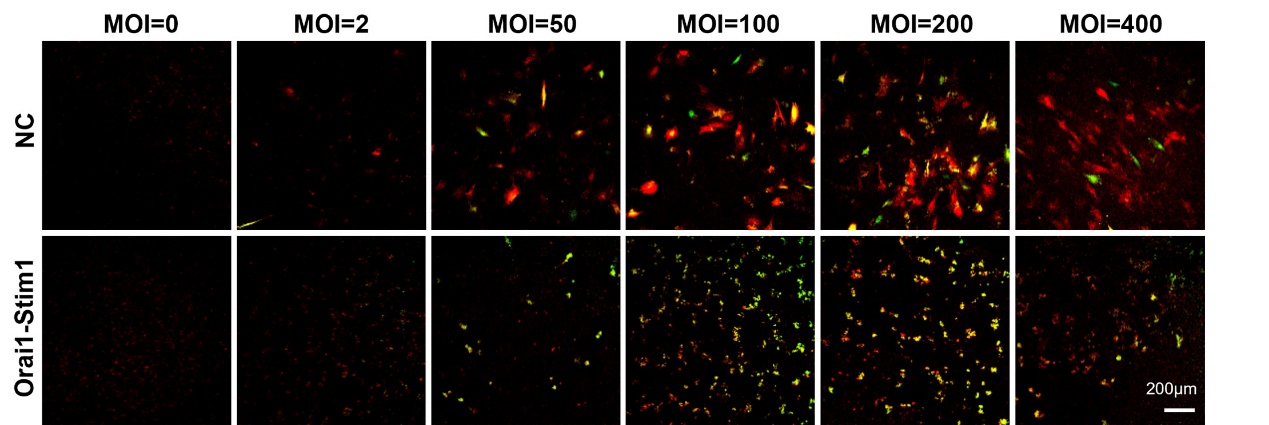


**Figure S5.** Determination of the optimal multiplicity of infection (MOI) for lentivirus transfection, Orai1-GFP, Stim1-mCherry.


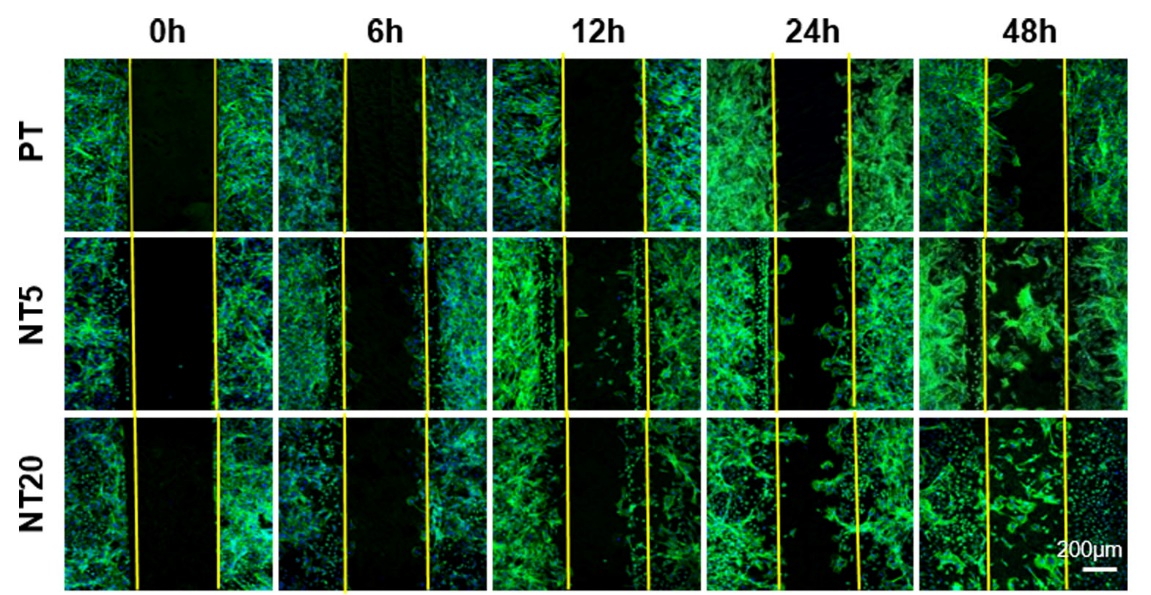


**Figure S6.** Influence of Ti nanotopographies on collective cell migration at 0h,

6h, 12h, 24h, 48h. Green-F-actin; Blue-nuclei.


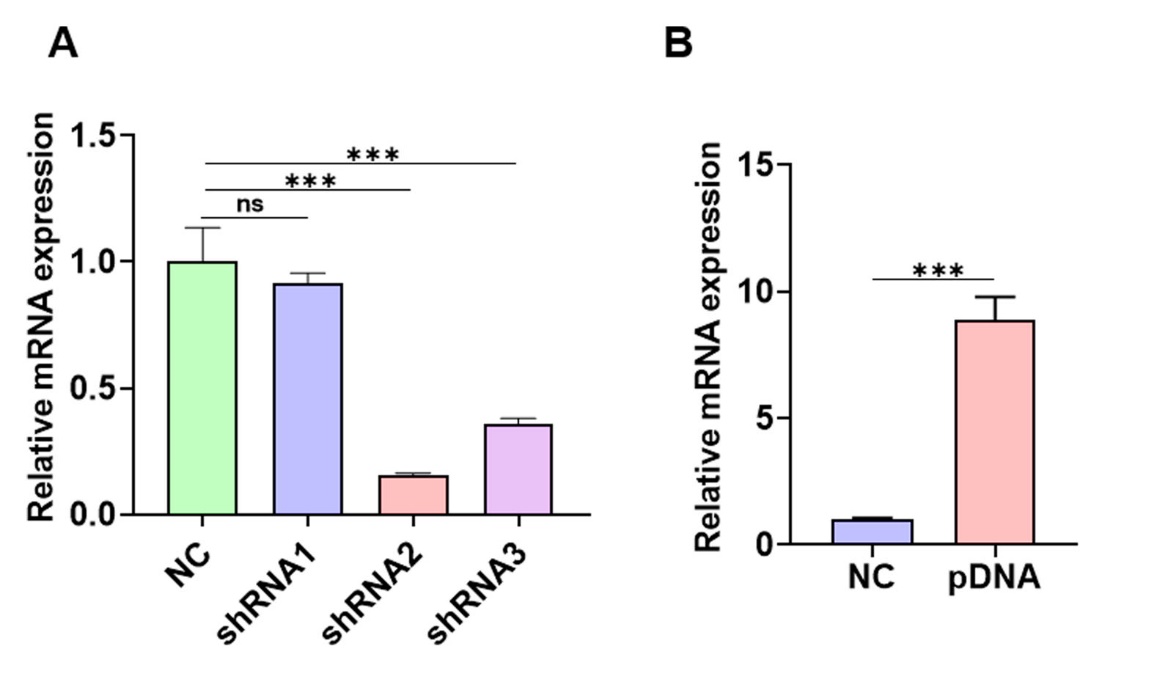


**Figure S7.** Screening of stim1 knockdown within three guarantees and validation of RNA levels after stim1 knockdown and overexpression. (A) The stim1 expression determined by RT-qPCR after been knockdown by shRNA (n = 5). (B) The stim1 expression determined by RT-qPCR after been overpressed (n = 6). Data are presented as mean ± SEM, One-way ANOVA, ns, not significant, ***P ≤ 0.001.


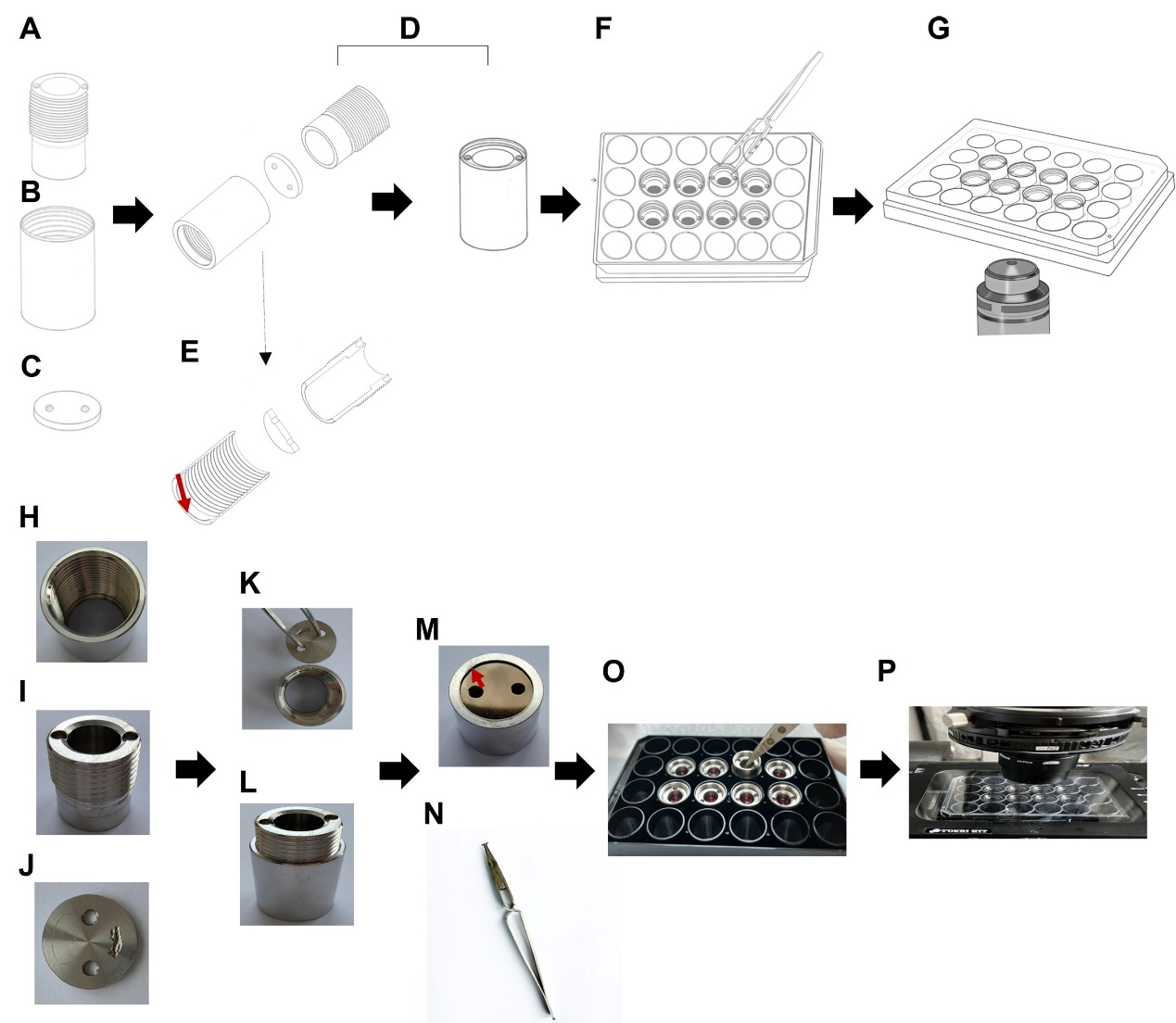


**Figure S8.** The diagram and the physical image of the holder used to assist in confocal imaging of live-cells. (A) The diagram of screw. (B) The diagram of nut. (C) The diagram of Ti plate (diameter = 13mm) with two holes of which the diameter was 2 mm. (D) Inverting the Ti plate seeded with mBMSCs into the holder and screwing the screw into the retainer to secure the titanium plate to form a wholepiece one. (E) The profile of the holder with the sample, the red arrow showed the step was 0.45mm thick and was designed to support the Ti specimen so as the cells on the specimen were in the confocal focusing range without any pressure. (F) Several holders were inserted into 0# 24 Well Glass Bottom Black Plate containing culture medium with a reverse pinching forceps. (G) 0# 24 Well Glass Bottom Black Plate was placed in the live-cell incubation chamber mounted on a live-cell workstation and live-cell time-lapse imaging was performed on an inverted Nikon A1Rsi laser scanning confocal microscope. (H) The nut made of stainless steel. (I) The screw made of stainless steel. (J) The round Ti plate with two holes. (K) Inverting the Ti plate seeded with mBMSCs into the holder. (L) Screwing the screw into the retainer to secure the titanium plate to form a wholepiece one. (M) After the Ti plate was fixed, the bottom of the holder was exhibited, and the arrow showed the 0.45 mm thick step. (N) The reverse pinching forceps with a protruding beak which was used to place or remove the holders from the confocal plate. (O) Several holders were inserted into 0# 24 Well Glass Bottom Black Plate containing culture medium with a reverse pinching forceps. (P) 0# 24 Well Glass Bottom Black Plate was placed in the live-cell incubation chamber mounted on a live-cell workstation and live-cell time-lapse imaging was performed on an inverted Nikon A1Rsi laser scanning confocal microscope.


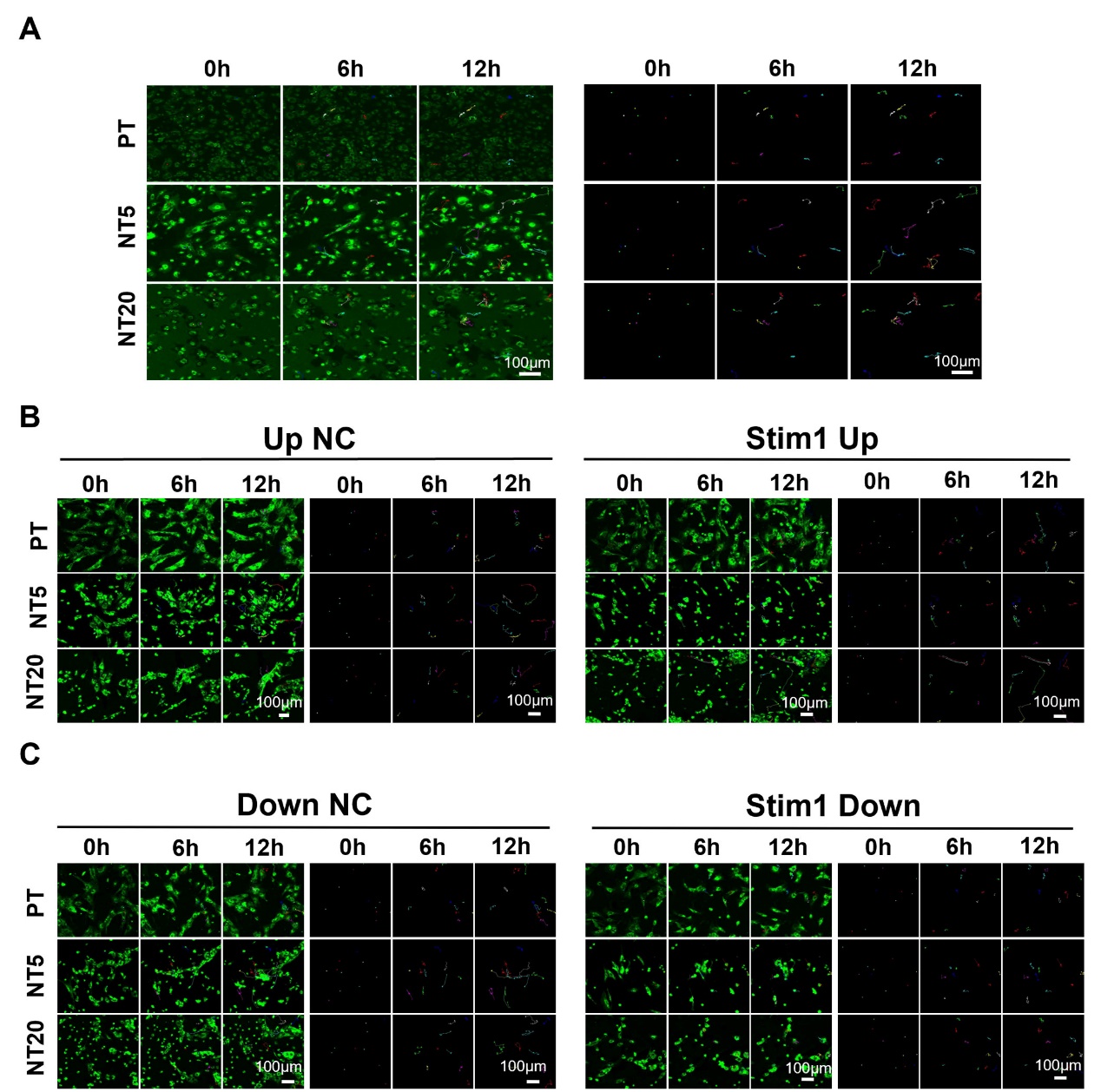


**Figure S9.** The live-cell time-lapse imaging and single-cell migration trajectories on different Ti surfaces. (A) The live-cell imaging with single-cell migration trajectories at 0h, 6h and 12h on different surfaces and the extracted cell trajectories at the three time points. (B, C) The live-cell imaging with single-cell migration trajectories after overexpression and knockdown of stim1 at 0h, 6h and 12h on the three surfaces and the extracted cell trajectories at the three time points.
